# Supplementary material for: Ketogenic Diet and Multiple Health Outcomes: An Umbrella Review of Meta-Analysis
Source: Nutrients. 2023 Sep 27;15(19):4161. doi: 10.3390/nu15194161 (PMC10574428; doi:10.3390/nu15194161)
Supplement: Supplementary file 1 [file nutrients-15-04161-s001.zip › nutrients-2576153-supplementary.pdf]

Supplementary methods. Search strategy for PubMed, Embase, and the Cochrane Database of Systematic Reviews

Search strategy for PubMed.

No.	Description or domain	Search term
1	Search terms for ketogenic diet (title/abstract)	"ketogenic diet" OR "very low-carbohydrate diet, keto-adaptation" OR ketosis OR "high fat diet" OR "low carbohydrate diet" OR "carbohydrate-restricted diet" OR VLCD OR KD
2	MeSH terms for unsaturated fatty acids	"Diet, Ketogenic"[Mesh]
3	Search terms for meta-analyses (title/abstract)	meta-analy* OR meta analy* OR metaanaly* OR meta-regression OR meta synthesis
4	MeSH terms for meta-analyses	Meta-Analysis[Publication Type] OR "Meta-Analysis as Topic"[Mesh]
5	Domain for ketogenic diet	1 OR 2
6	Domain for meta-analyses	3 OR 4
7	Total search string	5 AND 6

Search strategy for Embase.

No.	Description or domain	Search term
1	Search terms for ketogenic diet (title, abstract, and author keywords)	"ketogenic diet" OR "very low-carbohydrate diet, keto-adaptation" OR ketosis OR "high fat diet" OR "low carbohydrate diet" OR "carbohydrate-restricted diet" OR VLCD OR KD
2	MeSH terms for ketogenic diet	'Diet, Ketogenic'/exp
3	Search terms for meta-analyses (title, abstract, and author keywords)	meta-analy* OR meta analy* OR metaanaly* OR meta-regression OR meta synthesis
4	MeSH terms for meta-analyses	'meta analysis'/exp
5	Domain for unsaturated fatty acids	1 OR 2
6	Domain for meta-analyses	3 OR 4
7	Total search string	5 AND 6

Search strategy for The Cochrane Database of Reviews.

No.	Description or domain	Search term

1	Search terms for ketogenic diet (title, abstract, keyword)	"ketogenic diet" OR "very low-carbohydrate diet, keto-adaptation" OR ketosis OR "high fat diet" OR "low carbohydrate diet" OR "carbohydrate-restricted diet" OR VLCD OR KD
2	MeSH terms for ketogenic diet	"Diet, Ketogenic"[Mesh descriptor, explode all trees]
3	Search terms for meta-analyses (title, abstract, keyword)	meta-analy* OR meta analy* OR metaanaly* OR meta-regression OR meta synthesis
4	MeSH terms for meta-analyses	"Meta-Analysis as Topic"[Mesh descriptor, explode all trees]
5	Domain for ketogenic diet	1 OR 2
6	Domain for meta-analyses	3 OR 4
7	Total search string	5 AND 6

Supplemental Table S1 Quantitative synthesis and evidence grading for meta-analyses of diet intervention for clinical and non-clinical health problems

Study, year (ref)	Study disease or population	Intervention	Outcomes	No. of studies (participants, n)	Original effect metrics	Random-effects summary estimate (95% CI)	Random-effects ***P***	I^2, %	95% Prediction interval	Egger's test> ***P***	Largest study estimate (95% CI)	Significant studies		Grading
												**O/E**	***P***	
Castellana et al. 2020 [1]	Overweight, obese	Ketogenic phase < 4 weeks	Body weight	6 (384)	MD	-1.698 (-10.314,6.917)	0.699	0.0	(-13.903,10.506)	0.017	-1.45 (-13.04,10.13)	0/5.58	-	NS
		Ketogenic phase≥ 4 weeks	Body weight	6 (211)	MD	-2.962 (-13.979,8.054)	0.598	0.0	(-18.568,12.643)	0.019	-1.77 (-17.72,14.18)	0/5.62	-	NS
		VLCKD	Body weight	3 (222)	MD	-0.803 (-11.434,9.829)	0.882	0.0	(-69.726,68.12)	0.095	-0.54 (-15.61,14.54)	0/0.71	-	NS
		Ketogenic phase < 4 weeks	BMI	5 (312)	MD	-2.133 (-12.617,8.351)	0.690	0.0	(-19.157,14.89)	0.038	-2 (-15.88,11.87)	0/4.88	-	NS
		Ketogenic phase≥4 weeks	BMI	5 (145)	MD	-3.588 (-18.241,11.065)	0.631	0.0	(-27.381,20.205)	0.006	-2.08 (-19.72,15.56)	0/4.87	-	NS
		Ketogenic phase 4 weeks	WC	5 (312)	MD	-1.779 (-11.744,8.187)	0.726	0.0	(-17.96,14.403)	0.009	-1.48 (-13.17,10.21)	0/4.6	-	NS
		Ketogenic phase 4 weeks	WC	5 (145)	MD	-4.057 (-18.704,10.590)	0.587	0.0	(-27.84,19.726)	0.014	-2.53 (-22.82,17.75)	0/4.98	-	NS
		VLCKD	RR of discontinuation	3 (222)	RR	0.955 (0.610,1.495)	0.841	0.0	(0.052,17.408)	0.885	1.03 (0.62,1.7)	0/0.15	-	NS
Choi et al. 2020 [2]	Overweight or obese,	KD	BMI	2 (73)	SMD	-0.803 (-3.243,1.636)	0.519	94.8	-	0.000	0.42 (-0.23,1.06)	1/0.21	0.20	NS

	non-type 2 diabetes													
		KD	Body weight	3 (219)	SMD	-0.532 (-1.627,0.563)	0.341	89.9	(-14.081,13.017)	0.658	-0.14 (-0.46,0.19)	1/0.19	0.18	NS
		KD	Diastolic blood pressure	2 (184)	SMD	-0.288 (-0.996,0.420)	0.425	74.5	-	0.000	-0.6 (-0.94,-0.27)	1/0.68	1.00	NS
		KD	Fasting glucose	6 (290)	SMD	-0.348 (-0.764,0.069)	0.102	54.6	(-1.528,0.833)	0.425	-0.27 (-0.6,0.05)	1/0.48	0.39	NS
		KD	Fasting insulin	3 (200)	SMD	-0.150 (-0.985,0.684)	0.724	79.9	(-9.968,9.667)	0.934	-0.19 (-0.51,0.14)	2/0.21	0.01	NS
		KD	HDL	4 (235)	SMD	0.067 (-0.389,0.522)	0.774	52.4	(-1.672,1.805)	0.786	0.04 (-0.28,0.37)	0/0.2	-	NS
		KD	LDL	4 (235)	SMD	0.349 (0.087,0.610)	0.009	0.0	(-0.225,0.922)	0.228	0.25 (-0.08,0.57)	0/0.33	-	Weak
		KD	Serum creatinine	3 (223)	SMD	0.244 (-0.208,0.697)	0.290	52.5	(-4.481,4.969)	0.578	0.07 (-0.26,0.39)	1/0.16	0.15	NS
		KD	Systolic blood pressure	2 (184)	SMD	-0.331 (-1.069,0.406)	0.379	76.6	-	0.000	-0.66 (-0.99,-0.33)	1/0.78	1.00	NS
		KD	TC	4 (235)	SMD	0.381 (0.083,0.679)	0.012	11.6	(-0.435,1.197)	0.256	0.18 (-0.15,0.5)	2/0.27	0.02	Weak
		KD	TG	4 (235)	SMD	-0.175 (-0.458,0.109)	0.227	57.9	(-1.266,0.917)	0.029	-0.4 (0.02,0.24)	1/0.53	0.44	NS
		KD	WC	3 (219)	SMD	-0.700 (-1.847,0.447)	0.232	90.5	(-14.912,13.513)	0.584	-0.19 (-0.52,0.13)	1/0.22	0.20	NS
	Overweight or obese, type 2 diabetes	KD	C-reactive protein	3 (183)	SMD	0.081 (-0.209,0.371)	0.585	0.0	(-1.799,1.96)	0.939	0.09 (-0.27,0.46)	0/0.16	-	NS
		KD	Diastolic blood pressure	4 (233)	SMD	0.007 (-0.263,0.278)	0.958	0.0	(-0.586,0.601)	0.564	0.09 (-0.28,0.45)	0/0.22	-	NS
		KD	Systolic blood pressure	4 (233)	SMD	0.058 (-0.200,0.317)	0.657	0.0	(-0.508,0.625)	0.920	0.08 (-0.29,0.44)	0/0.21	-	NS
Muscogiuri et al. 2021 [3]	Obesity	VLCKD(control)	BMI	9 (210)	MD	-7.037 (-20.076,6.003)	0.290	0.0	(-22.769,8.695)	0.094	-7.61 (-36.11,20.9)	0/9	-	NS

	VLCKD(control)	Body weight	10 (198)	MD	-3.031 (-8.810,2.747)	0.304	0.0	(-9.83,3.767)	0.003	-1.69 (-13.45,10.07)	0/7.14	-	NS
	VLCKD(control)	FFM	9 (242)	MD	-0.415 (-3.380,2.550)	0.784	0.0	(-3.992,3.162)	0.864	-0.6 (-6.87,5.68)	0/1.82	-	NS
	VLCKD(control)	FM	11 (271)	MD	-2.158 (-8.058,3.742)	0.473	0.0	(-8.968,4.652)	0.047	-0.56 (-9.43,8.31)	0/1.9	-	NS
	VLCKD(control)	Glycemia	10 (275)	MD	-1.586 (-5.541,2.369)	0.432	0.0	(-6.239,3.067)	0.025	-1.49 (-9.51,6.54)	0/6.67	-	NS
	VLCKD(control)	HDL	11 (323)	MD	0.694 (-2.605,3.993)	0.680	0.0	(-3.113,4.502)	0.168	0.36 (-7.17,7.9)	0/1.28	-	NS
	VLCKD(control)	HOMA-IR	8 (183)	MD	-2.575 (-8.194,3.045)	0.369	0.0	(-9.591,4.441)	0.001	-1.99 (-11.36,7.38)	0/6.37	-	NS
	VLCKD(control)	HbA1c	5 (241)	MD	-1.041 (-9.244,7.162)	0.804	0.0	(-14.36,12.279)	0.065	-0.06 (-10.25,10.13)	0/0.26	-	NS
	VLCKD(control)	LDL	11 (323)	MD	-0.898 (-4.037,2.242)	0.575	0.0	(-4.521,2.726)	0.137	-0.48 (-6.62,5.66)	0/1.84	-	NS
	VLCKD(control)	TC	11 (323)	MD	-0.991 (-4.264,2.283)	0.553	0.0	(-4.768,2.787)	0.002	-0.23 (-6.18,5.71)	0/0.85	-	NS
	VLCKD(control)	TG	11 (324)	MD	-2.110 (-6.723,2.504)	0.370	0.0	(-7.435,3.216)	0.098	-2.09 (-11.76,7.58)	0/9.61	-	NS
	VLCKD(control)	WC	11 (239)	MD	-3.575 (-10.082,2.933)	0.282	0.0	(-11.086,3.937)	0.001	-1.96 (-11.26,7.33)	0/8.81	-	NS
	VLCKD	BMI	5 (394)	MD	-0.256 (-8.439,7.927)	0.951	0.0	(-13.543,13.031)	0.007	-0.03 (-8.35,8.28)	0/0.25	-	NS
	VLCKD	Body weight	6 (426)	MD	-1.424 (-10.128,7.280)	0.748	0.0	(-13.754,10.906)	0.007	-0.98 (-10.89,8.94)	0/3.15	-	NS
	VLCKD	FFM	3 (127)	MD	1.749 (-13.059,16.557)	0.817	0.0	(-94.249,97.747)	0.833	2.89 (-15.16,20.94)	0/2.89	-	NS
	VLCKD	FM	3 (127)	MD	-0.087 (-8.371,8.197)	0.984	0.0	(-53.794,53.62)	0.015	0.01 (-8.3,8.33)	0/0.15	-	NS

		VLCKD	Glycemia	3 (198)	MD	0.251 (-21.008,21.509)	0.982	0.0	(-137.566,138.067)	0.217	1.87 (-22.16,25.9)	0/2.92	-	NS
		VLCKD	HDL	3 (198)	MD	3.302 (-19.661,26.264)	0.778	0.0	(-145.562,152.165)	0.348	3.1 (-31.71,37.91)	0/3	-	NS
		VLCKD	HOMA-IR	2 (145)	MD	-16.806 (-151.859,118.247)	0.807	0.0	-	0.000	-17.42 (-195.46,160.62)	0/2	-	NS
		VLCKD	HbA1c	3 (198)	MD	1.160 (-27.578,29.899)	0.937	0.0	(-185.149,187.47)	0.138	2.61 (-27.78,33)	0/3	-	NS
		VLCKD	LDL	3 (198)	MD	-0.855 (-16.710,15.001)	0.916	0.0	(-103.644,101.935)	0.090	0.36 (-18.5,19.22)	0/0.37	-	NS
		VLCKD	TC	3 (198)	MD	-3.245 (-25.569,19.079)	0.776	0.0	(-147.972,141.482)	0.522	-3.31 (-40.08,33.46)	0/3	-	NS
		VLCKD	TG	3 (198)	MD	-7.423 (-55.623,40.778)	0.763	0.0	(-319.9,305.055)	0.343	-7.28 (-80.72,66.15)	0/3	-	NS
		VLCKD	WC	4 (216)	MD	-4.237 (-24.936,16.462)	0.688	0.0	(-49.678,41.203)	0.006	-3.61 (-25.28,18.06)	0/3.98	-	NS
Cao et al. 2021 [4]	Endurance athletes	K-LCHF	HRmax	8 (128)	SMD	0.140 (-0.352,0.632)	0.577	52.3	(-1.255,1.535)	0.798	0.74 (-0.09,1.57)	1/1.03	-	NS
		K-LCHF	RER	8 (135)	SMD	-1.811 (-2.492,-1.13)	0.000	58.7	(-3.805,0.183)	0.023	-1.57 (-2.51,-0.64)	6/3.08	0.06	Weak
		K-LCHF	RPE	6 (112)	SMD	0.138 (-0.582,0.859)	0.707	70.2	(-2.186,2.462)	0.133	-1.08 (-1.95,-0.22)	2/1.38	0.63	NS
		K-LCHF	TTE	3 (48)	SMD	-0.126 (-0.658,0.406)	0.643	0.0	(-3.575,3.323)	0.250	0.04 (-0.76,0.84)	0/0.15	-	NS
		K-LCHF	VO_2max	10 (141)	SMD	-0.056 (-0.357,0.245)	0.716	0.0	(-0.41,0.299)	0.377	-0.07 (-0.87,0.73)	0/0.51	-	NS
Bueno et al. 2013 [5]	CV risk factor	VLCKD	Body weight	5 (588)	MD	-0.373 (-9.6,8.853)	0.937	0.0	(-15.355,14.609)	0.451	-0.49 (-13.05,12.07)	0/1.53	-	NS
		VLCKD	DBP	4 (397)	MD	0.193 (-9.148,9.535)	0.968	0.0	(-20.315,20.701)	0.158	0.25 (-11.85,12.35)	0/0.42	-	NS
		VLCKD	HDL	4 (355)	MD	0.555 (-9.658,10.768)	0.915	0.0	(-21.866,22.976)	0.059	0 (-11.93,11.93)	0/0.2	-	NS
		VLCKD	LDL	4 (355)	MD	0.362 (-9.09,9.813)	0.940	0.0	(-20.387,21.111)	0.436	0 (-11.93,11.93)	0/0.2	-	NS
		VLCKD	SBP	4 (397)	MD	-0.320 (-9.528,8.888)	0.946	0.0	(-20.535,19.895)	0.281	-1.13 (-16.1,13.83)	0/2.95	-	NS
		VLCKD	TG	4 (355)	MD	-1.055 (-11.486,9.377)	0.843	0.0	(-23.955,21.846)	0.405	-1.11 (-15.95,13.74)	0/2.88	-	NS
	Healthy people	VLCKD	Body weight	5 (562)	MD	-0.504 (-7.132,6.123)	0.881	0.0	(-11.266,10.257)	0.283	-0.4 (-9.01,8.21)	0/1.06	-	NS
		VLCKD	DBP	5 (658)	MD	0.073 (-9.107,9.254)	0.988	0.0	(-14.833,14.98)	0.268	-0.34 (-14.3,13.61)	0/0.94	-	NS
		VLCKD	HDL	5 (639)	MD	2.416 (-13.579,18.411)	0.767	0.0	(-23.555,28.388)	0.110	1.96 (-20.93,24.85)	0/4.94	-	NS
		VLCKD	LDL	5 (639)	MD	0.679 (-8.963,10.321)	0.890	0.0	(-14.977,16.335)	0.125	0 (-13.58,13.58)	0/0.25	-	NS

		VLCKD	SBP	5 (605)	MD	-0.285 (-7.715,7.144)	0.940	0.0	(-12.348,11.778)	0.285	-0.27 (-10.65,10.1)	0/0.66	-	NS
		VLCKD	TG	5 (639)	MD	-1.224 (-12.794,10.345)	0.836	0.0	(-20.01,17.562)	0.498	-0.87 (-16.73,14.99)	0/3.22	-	NS
	Type 2 Diabetes Mellitus	VLCKD	DBP	2 (243)	MD	0.766 (-18.777,20.309)	0.939	0	-	0.000	0.16 (-23.68,24)	0/0.15	-	NS
		VLCKD	SBP	2 (243)	MD	0.403 (-21.668,22.474)	0.971	0	-	0.000	0.99 (-22.71,24.7)	0/1.69	-	NS
		VLCKD	TG	3 (264)	MD	-0.676 (-9.425,8.072)	0.880	0	(-57.393,56.04)	0.255	-0.63 (-10.80,9.53)	0/1.07	-	NS
Gibson et al. 2015 [6]	-	KD	Desire to eat	4 (112)	WMD	-0.569 (-6.793,5.656)	0.858	0.0	(-14.233,13.095)	0.306	0.23 (-10.27,10.73)	0/0.3	-	NS
		KD	Fullness/Satiety	4 (112)	WMD	0.579 (-5.113,6.272)	0.842	0.0	(-11.917,13.075)	0.207	0.29 (-7.94,8.51)	0/0.35	-	NS
		KD	Hunger	5 (246)	WMD	-0.645 (-6.247,4.958)	0.822	0.0	(-9.742,8.452)	0.394	-0.09 (-10.48,10.3)	0/0.28	-	NS
		KD	Prospective consumption	2 (67)	WMD	-0.109 (-8.084,7.866)	0.979	0.0	-	0.000	-0.24 (-10.74,10.27)	0/0.16	-	NS
Sourbron et al. 2020 [7]	Pediatric seizures	KD and/or MAD	≥50% seizure reduction	5 (374)	RR	4.776 (2.976,7.665)	< 0.001	0.0	(2.216,10.295)	0.401	4.51 (2.03,10.01)	5/4.44	1.00	Weak
Yang et al. 2021 [8]	Cancer	KD	Fasting glucose	4 (100)	SMD	-0.402 (-1.232,0.427)	0.342	82.3	(-4.157,3.352)	0.185	-1.49 (-2.06,-0.91)	1/2.53	-	NS
		KD	Fasting insulin	3 (90)	SMD	0.107 (-1.331,1.545)	0.884	89.1	(-17.665,17.878)	0.033	-0.96 (-1.59,-0.32)	2/0.85	0.20	NS
		KD	HDL	3 (91)	SMD	-0.185 (-0.975,0.604)	0.646	69.2	(-9.143,8.773)	0.370	0.49 (-0.12,1.1)	0/0.33	-	NS
		KD	LDL	3 (91)	SMD	0.211 (-0.206,0.629)	0.321	0.0	(-2.497,2.92)	0.383	0.08 (-0.52,0.68)	0/0.15	-	NS
		KD	TC	3 (115)	SMD	0.248 (-0.170,0.666)	0.245	0.0	(-2.463,2.96)	0.168	0.11 (-0.49,0.71)	0/0.16	-	NS
		KD	TG	3 (91)	SMD	0.152 (-0.481,0.784)	0.638	53.0	(-6.442,6.745)	0.445	-0.35 (-0.95,0.26)	0/0.24	-	NS
		KD	Adverse events	2 (329)	RR	1.263 (0.301,5.302)	0.750	81.2	-	0.000	0.65 (0.35,1.2)	0/0.65	-	NS
		KD	Ketosis	2 (62)	RR	3.578 (1.361,9.404)	0.010	0.0	-	0.000	5.65 (1.45,22.08)	1/1.37	-	Weak
Whittaker et al. 2022 [9]	Healthy adult males	HP-KD	Total testosterone, resting	2 (19)	SMD	-1.040 (-1.729,-0.351)	0.003	0.0	-	0.000	-1.23 (-2.21,-0.26)	1/0.53	0.46	Weak

		KD≥3weeks	Cortisol, resting	4 (54)	SMD	-0.073 (-0.526,0.379)	0.751	11.0	(-1.268,1.121)	0.920	-0.02 (-0.74,0.69)	0/0.2	-	NS
		KD≥3weeks	Total testosterone, resting	5 (73)	SMD	0.049 (-0.332,0.430)	0.801	0.0	(-0.57,0.668)	0.031	0.51 (-0.22,1.24)	0/0.47	-	NS
		KD < 3weeks	Cortisol, resting	8 (78)	SMD	0.278 (-0.053,0.609)	0.099	0.0	(-0.135,0.692)	0.946	0.18 (-0.56,0.93)	0/0.43	-	NS
		KD < 3weeks	Total testosterone, resting	2 (19)	SMD	-1.040 (-1.729,-0.351)	0.003	0.0	-	0.000	-1.23 (-2.21,-0.26)	1/0.53	0.46	Weak
		MP- KD	Total testosterone, resting	5 (73)	SMD	0.049 (-0.332,0.430)	0.801	0.0	(-0.57,0.668)	0.031	0.51 (-0.22,1.24)	0/0.47	-	NS
Martin-McGill et al. 2020 [10]	Drug-resistant epilepsy	KD	≥50% seizure reduction: adults	2 (141)	RR	4.802 (0.344,67.046)	0.243	62.3	-	0.000	1.54 (0.27,8.7)	1/0.33	0.31	NS
		KD	≥50% seizure reduction: children	4 (385)	RR	5.641 (3.392,9.38)	< 0.001	0.0	(1.847,17.225)	0.638	4.51 (2.03,10.01)	4/3.81	1	Weak
		KD	Seizure freedom: children	4 (385)	RR	2.721 (0.992,7.461)	0.052	0.0	(0.297,24.917)	0.431	2.93 (0.63,13.65)	0/3.14	-	NS
		KD	Treatment withdrawal: children	5 (425)	RR	1.016 (0.695,1.486)	0.934	0.000	(0.549,1.883)	0.071	0.81 (0.49,1.36)	0/0.41	-	NS

Zhou et al. 2022 [11]	Type 2 diabetes	KD	BMI	7 (535)	MD	-0.433 (-7.434,6.569)	0.904	0.0	(-9.615,8.75)	0.220	-0.33 (-12.73,12.07)	0/0.84	-	NS
		KD	Body weight	7 (445)	MD	-0.861 (-7.790,6.067)	0.808	0.0	(-9.948,8.226)	0.058	-0.32 (-12.69,12.06)	0/0.71	-	NS
		KD	Fasting glucose	5 (399)	MD	0.057 (-0.242,0.356)	0.708	39.4	(-0.758,0.873)	0.665	0.25 (-0.12,0.62)	0/0.47	-	NS
		KD	Fasting insulin	5 (332)	MD	-0.829 (-8.659,7.002)	0.836	0.0	(-13.543,11.886)	0.388	-0.37 (-12.83,12.09)	0/0.64	-	NS
		KD	HDL	7 (458)	MD	0.283 (0.098,0.468)	0.003	0.0	(0.04,0.525)	0.935	0.47 (0.1,0.84)	1/1.25	-	Weak
		KD	HOMA-IR	5 (367)	MD	-0.787 (-8.899,7.325)	0.849	0.0	(-13.959,12.385)	0.205	-0.36 (-12.81,12.09)	0/0.68	-	NS
		KD	HbA1c	6 (343)	MD	-1.581 (-10.104,6.942)	0.716	0.0	(-13.654,10.493)	0.584	-1.25 (-16.84,14.34)	0/3.87	-	NS
		KD	LDL	7 (458)	MD	0.102 (-0.082,0.285)	0.277	0.0	(-0.139,0.342)	0.239	0.08 (-0.28,0.45)	0/0.37	-	NS
		KD	TC	4 (365)	MD	0.003 (-0.203,0.209)	0.978	0.0	(-0.45,0.455)	0.821	0.07 (-0.29,0.44)	0/0.22	-	NS
		KD	TG	7 (458)	MD	-0.367 (-0.553,-0.181)	0.000	0.0	(-0.611,-0.123)	0.525	-0.2 (-0.57,0.17)	1/0.51	0.41	Weak
		KD	WC	5 (467)	MD	-0.775 (-11.063,9.513)	0.883	0.0	(-17.479,15.929)	0.022	-0.42 (-17.19,16.35)	0/0.93	-	NS
Pizzo et al. 2022 [12]	Pediatric seizure	KD	≥50% seizure reduction	7 (413)	OR	4.641 (2.307,9.335)	< 0.001	43.5	(0.754,28.569)	0.448	8.31 (3.01,22.93)	5/6.81	-	Weak
Amini et al. 2022 [13]	Overweight	KD	BMI	5 (178)	WMD	-0.145 (-5.536,5.245)	0.958	0.0	(-8.898,8.607)	0.070	0 (-8.11,8.11)	0/0.25		NS
		KD	Body weight	5 (178)	WMD	-0.623 (-7.008,5.761)	0.848	0.0	(-10.99,9.744)	0.043	0 (-8.11,8.11)	0/0.25	-	NS
		KD	FFM	3 (143)	WMD	0.081 (-6.958,7.121)	0.982	0.0	(-45.555,45.717)	0.687	0.15 (-8.89,9.2)	0/0.18	-	NS
		KD	FM	5 (178)	WMD	0.019 (-5.855,5.893)	0.995	0.0	(-9.519,9.557)	0.244	0.19 (-7.99,8.37)	0/0.3	-	NS
		KD	WC	2 (124)	WMD	-0.910 (-14.311,12.490)	0.894	0.0	-	0.000	-0.57 (-16.81,15.67)	0/0.46	-	NS
	Obese cardiovascular risk factor	KD	Body weight	3 (158)	WMD	-0.356 (-10.325,9.614)	0.944	0.0	(-64.987,64.275)	0.283	0.5 (-14.74,15.73)	0/0.49	-	NS

	Overweight, hyperlipidemic	KD	Body weight	2 (239)	WMD	0.244 (-23.469,23.957)	0.984	0.0	-	0.000	0.9 (-24.53,26.33)	0/1.54	-	NS
	Healthy people	KD	LBM	2 (33)	WMD	-0.733 (-6.139,4.673)	0.790	0.0	-	0.000	-0.64 (-6.9,5.63)	0/0.2	-	NS
		KD	VAT	2 (33)	WMD	-1.169 (-7.562,5.223)	0.720	0.0	-	0.000	-1.1 (-8.72,6.51)	0/0.42	-	NS
Taftian et al. 2022 [14]	Cancer	KD	BMI	4 (186)	WMD	-1.401 (-12.053,9.251)	0.797	0.0	(-24.784,21.982)	0.034	-0.85 (-17.47,15.76)	0/1.38	-	NS
		KD	Body weight	7 (246)	WMD	-0.215 (-4.529,4.100)	0.922	0.0	(-5.873,5.444)	0.009	0.54 (-6.01,7.1)	0/0.97	-	NS
Zhang et al. 2022 [15]	CDKL5-related epilepsy	KD	Clinical responder rate	11 (183)	OR	0.626 (0.342,1.147)	0.130	45.6	(0.133,2.942)	0.011	1.42 (0.96,2.1)	2/0.92	0.23	NS
		KD	Definite responder rate	3 (24)	OR	0.225 (0.070,0.721)	0.012	0.0	(0,427.408)	0.639	0.2 (0.04,0.91)	1/1.04	-	Weak

BMI, body mass index; DBP, diastolic blood pressure; FFM, fat-free mass; FM, fat mass; HDL, high density lipoprotein; HOMA-IR, homeostatic model assessment index of insulin resistance; HbA1c, glycated hemoglobin; HRmax, maximal heart rate during exercise; LBM, lean body mass; LDL, low density lipoprotein; RER, respiratory exchange rate; RPE, perceived exertion; SBP, systolic blood pressure; TTE, time to exhaustion; TC, total cholesterol; TG, triglycerides; VAT, visceral adipose tissue; VO_2max, maximum oxygen uptake; WC, waist circumference; KD, ketogenic diet; VLCKD, very-low-carbohydrate ketogenic diets; K-LCHF, ketogenic low-carbohydrate, high-fat; MAD, modified Atkins diet; HP, high-protein; MP, moderate-protein; MD, mean difference; O, observed number of studies with positive finding; ref, reference; SMD, standardized mean difference; WMD, weighted mean difference; OR, odds ratio; RR, relative risk.

Supplemental Table S2 Quantitative synthesis and evidence grading for meta-analyses of combination of diet and physical activity interventions for clinical and non-clinical health problems

Study, year (ref)	Study disease or population	Intervention	Outcomes	No. of studies (participants, n)	Original effect metrics	Random-effects summary estimate (95% CI)	Random-effects *P*	I^2, %	95% Prediction interval	Egger's test> *P*	Largest study estimate (95% CI)	Significant studies		Grading
												O/E	*P*	
Lee et al. 2021 [16]	Overweight and obese	Exercise, KD	BMI	5 (136)	SMD	-0.122 (-0.532,0.287)	0.557	37.6	(-1.245,1.0)	0.257	-0.24 (-0.73,0.26)	1/0.31	0.28	NS
		Exercise, KD	Body fat mass	2 (49)	SMD	-0.146 (-0.751,0.459)	0.636	0.0	-	0.000	0.07 (-0.7,0.82)	0/0.10	-	NS
		Exercise, KD	Body weight	5 (136)	SMD	-0.301(-0.545,-0.056)	0.016	3.64	(-0.745,0.143)	0.011	-0.47 (-0.35,0.22)	1/0.49718677	0.40766193	Weak
		Exercise, KD	Cardiorespiratory fitness (VO^2peak)	3 (87)	SMD	0.029 (-0.139,0.196)	0.738	10.3	(-1.544,1.602)	0.059	-0.01 (-0.5,-0.48)	0/0.15	-	NS
		Exercise, KD	Fasting glucose	4 (103)	SMD	-0.063 (-0.363,0.237)	0.682	0	(-0.721,0.596)	0.236	-0.01 (-0.4,0.39)	0/0.2	-	NS
		Exercise, KD	HDL	3 (74)	SMD	0.188 (-0.079,0.455)	0.167	0	(-1.543,1.92)	0.673	0.25 (-0.5,0.1)	0/0.19	-	NS
		Exercise, KD	LDL	3 (74)	SMD	0.736 (0.262,1.210)	0.002	0	(-2.336,3.809)	0.682	0.85 (0.1,1.6)	1/0.60	0.49	Weak
		Exercise, KD	Lean body mass	2 (49)	SMD	-0.739 (-2.406,0.929)	0.385	87.0	-	0.000	0.03 (-0.73,0.08)	1/0.1	0.1	NS
		Exercise, KD	TC	3 (74)	SMD	0.315 (-0.155,0.786)	0.189	3.0	(-2.875,3.506)	0.227	0 (-0.72,0.72)	0/0.15	-	NS
		Exercise, KD	TG	3 (74)	SMD	-0.443 (-0.912,0.026)	0.064	1.8	(-3.564,2.678)	0.081	-0.29 (-0.99,0.45)	1/0.2	0.19	NS

		Exercise, KD	WC	2 (58)	SMD	-0.781 (-1.760,0.198)	0.118	69.2	-	0.000	-0.3 (-1.02,0.42)	1/0.14	0.14	NS
Lee et al. 2021 [17]	-	Exercise, KD	Body mass	2 (39)	SMD	0.053 (-0.542,0.647)	0.862	0.0	-	0.000	0.28 (-0.51,1.07)	0/0.12	-	NS
Whittaker et al. 2022 [9]	Healthy adult males	HP-KD	Cortisol, 0 hr post-exercise	2 (14)	SMD	-0.052 (-1.461,1.357)	0.942	69.1	-	0.000	0.63 (-0.38,1.64)	0/0.18	-	NS
		KD, long-duration exercise	Cortisol, 0 hr post-exercise	6 (64)	SMD	0.705 (0.326,1.084)	< 0.001	0.0	(0.168,1.242)	0.532	0.52 (-0.23,1.28)	1/0.53	0.43	Weak
		KD, long-duration exercise	Cortisol, 1 hr post-exercise	4 (42)	SMD	0.650 (0.206,1.094)	0.004	0.0	(-0.324,1.625)	0.710	0.41 (-0.34,1.16)	1/0.3	0.27	Weak
		KD, short-duration exercise	Cortisol, 0 hr post-exercise	2 (17)	SMD	-0.671 (-1.370,0.028)	0.060	0.0	-	0.000	-0.6 (-1.46,0.26)	0/0.19	-	NS
		KD≥3weeks	Cortisol, 0 hr post-exercise	2 (19)	SMD	0.174 (-1.403,1.751)	0.829	81.3	-	0.000	-0.6 (-1.46,0.26)	0/0.2	-	NS
		KD < 3weeks	Cortisol, 0 hr post-exercise	6 (62)	SMD	0.500 (0.084,0.916)	0.018	13.1	(-0.288,1.288)	0.277	0.52 (-0.23,1.28)	1/0.52	0.42	Weak
		MP-KD	Cortisol, 0 hr post-exercise	6 (67)	SMD	0.488 (0.007,0.969)	0.047	39.5	(-0.759,1.736)	0.626	0.52 (-0.23,1.28)	1/0.55	0.44	Weak
		MP-KD < 3weeks & long-duration exercise	Cortisol, 2 hr post-exercise	3 (36)	SMD	0.818 (0.333,1.302)	0.001	0.0	(-2.324,3.959)	0.304	0.61 (-0.15,1.37)	2/0.36	0.04	Weak

Ashtary-Larky et al. 2022 [18]	Healthy people	Exercise, KD	BFP	4 (84)	WMD	-0.966 (-9.214,7.282)	0.818	0.0	(-19.073,17.14)	0.180	0.75 (-9.06,10.56)	0/0.62	-	NS
		Exercise, KD	BMI	3 (67)	WMD	-1.136 (-8.504,6.231)	0.762	0.0	(-48.9,46.628)	0.103	-0.73 (-10.49,9.03)	0/0.45	-	NS
		Exercise, KD	Body mass	11 (212)	WMD	-0.820 (-3.705,2.065)	0.577	0.0	(-4.15,2.51)	0.000	-0.69 (-6.71,5.33)	0/1.45	-	NS
		Exercise, KD	FFM	11 (212)	WMD	-0.639 (-3.308,2.029)	0.639	0.0	(-3.719,2.441)	0.006	-0.1 (-5.86,5.65)	0/0.57	-	NS
		Exercise, KD	FM	10 (192)	WMD	-1.126 (-4.584,2.333)	0.524	0.0	(-5.195,2.944)	0.003	-0.65 (-7.05,5.76)	0/1.22	-	NS

Supplemental Table S3. Efficacy outcome of epilepsy (%) according to different dietary regimens/patterns.

Study, year (ref)	Classification	Design	Intervention	Efficacy outcome		
				≥50%	≥90%	SF
KD						
Liu et al. 2019 [19]	Dravet syndrome	Retrospective	KD (*n* = 26)	3mo: 38.5% (10/26)<br>6mo: 34.6% (9/26)<br>12mo: 84.6% (22/26)	-	-
Liu et al. 2019 [20]	Dravet syndrome	Prospective	KD (*n* = 16)	3mo: 62.5% (10/16)<br>6mo: 75.0% (12/16)<br>12mo: 68.8% (11/16)	-	-
Ni et al. 2018 [21]	Dravet syndrome	Prospective	KD (*n* = 20)	3mo: 85.0% (17/20)<br>6mo: 85.0% (17/20)	-	-
Tian et al. 2018 [22]	Dravet syndrome	Retrospective	KD (*n* = 60)	3mo: 58.3% (35/60)<br>6mo: 41.7% (25/60)<br>12mo: 28.3% (17/60)	-	-
Dressler et al. 2015 [23]	Dravet syndrome	Retrospective	KD (*n* = 10)	3mo: 70.0% (7/10)<br>6mo: 60.0% (6/10)<br>12mo: 60.0% (6/10)	-	3mo: 10.0% (1/10)
Caraballo et al. 2011 [24]	Dravet syndrome	Retrospective	KD (*n* = 24)	-	-	-
Nabbout et al. 2011 [25]	Dravet syndrome	Prospective	KD (*n* = 15)	-	-	-
Caraballo et al. 2005 [26]	Dravet syndrome	Retrospective	KD (*n* = 20)	6mo: 65.0% (13/20)<br>12mo: 65.0% (13/20)	-	-
Nei et al. 2014 [27]	Epilepsy in adults and adolescents	Prospective	KD (*n* = 29)	9mo: 44.8% (13/29)		

Dressler et al. 2019 [28]	Infant epilepsy	Prospective	KD (*n* = 16)	3mo: 62.5% (10/16)	-	3mo: 62.5% (10/16)
Dressler et al. 2018 [29]	Infant epilepsy	Prospective	KD (*n* = 11)	3mo: 72.7% (8/11)	-	3mo: 27.3% (3/11)
Ismayilova et al. 2018 [30]	Infant epilepsy	Retrospective	KD (*n* = 29)	3mo: 24.1% (7/29)	-	3mo: 6.9% (2/29)
Wirrell et al. 2018 [31]	Infant epilepsy	Retrospective	KD (*n* = 26)	3mo: 61.5% (16/26)	-	3mo: 34.6% (9/26)
Yan et al. 2018 [21]	Infant epilepsy	Prospective	KD (*n* = 6)	3mo: 100.0% (6/6)	-	-
Zhang et al. 2018 [32]	Infant epilepsy	Prospective	KD (*n* = 2)	3mo: 50.0% (1/2)	-	3mo: 0.0% (0/2)
Sampaio et al. 2017 [33]	Infant epilepsy	Prospective	KD (*n* = 1)	3mo: 100.0% (1/1)	-	-
Kim et al. 2016 [34]	Infant epilepsy	Retrospective	KD (*n* = 37)	3mo: 48.6% (18/37)	-	3mo: 35.1% (13/37)
Wu et al. 2016 [35]	Infant epilepsy	Retrospective	KD (*n* = 6)	3mo: 33.3% (2/6)	-	3mo: 16.7% (1/6)
Dressler et al. 2015 [36]	Infant epilepsy	Retrospective	KD (*n* = 58)	3mo: 63.8% (37/58)	-	3mo: 34.5% (20/58)
Hirano et al. 2015 [37]	Infant epilepsy	Retrospective	KD (*n* = 5)	3mo: 100.0% (5/5)	-	3mo: 20.0% (1/5)
van der Louw et al. 2015 [38]	Infant epilepsy	Retrospective	KD (*n* = 25)	3mo: 56.0% (14/25)	-	-
Vehmeijer et al. 2015 [39]	Infant epilepsy	Retrospective	KD (*n* = 16)	3mo: 31.3% (5/16)	-	-
Caraballo et al. 2014 [40]	Infant epilepsy	Retrospective	KD (*n* = 1)	3mo: 100.0% (1/1)	-	3mo: 0.0% (0/1)
Pires et al. 2013 [41]	Infant epilepsy	Prospective	KD (*n* = 17)	3mo: 88.2% (15/17)	-	3mo: 64.7% (11/17)
Jung et al. 2012 [42]	Infant epilepsy	Retrospective	KD (*n* = 2)	3mo: 100.0% (2/2)	-	3mo: 50.0% (1/2)
Kumada et al. 2012 [43]	Infant epilepsy	Prospective	KD (*n* = 3)	3mo: 66.7% (2/3)	-	3mo: 66.7% (2/3)
Larson et al. 2012 [44]	Infant epilepsy	Retrospective	KD (*n* = 2)	3mo: 100.0% (2/2)	-	3mo: 50.0% (1/2)
Sharma et al. 2012 [45]	Infant epilepsy	Prospective	KD (*n* = 6)	3mo: 50.0% (3/6)	-	3mo: 50.0% (3/6)
Coppola et al. 2010 [46]	Infant epilepsy	Prospective	KD (*n* = 11)	3mo: 81.8% (9/11)	-	-
Tonekaboni et al. 2010 [47]	Infant epilepsy	Prospective	KD (*n* = 3)	3mo: 66.7% (2/3)	-	3mo: 33.3% (1/3)
Villeneuve et al. 2009 [48]	Infant epilepsy	Retrospective	KD (*n* = 5)	3mo: 20.0% (1/5)	-	

Dahlin et al. 2007 [49]	Infant epilepsy	Prospective	KD (*n* = 2)	3mo: 50.0% (1/2)	-	-
Eun et al. 2006 [50]	Infant epilepsy	Retrospective	KD (*n* = 10)	3mo: 60.0% (6/10)	-	3mo: 60.0% (6/10)
Dahlin et al. 2005 [51]	Infant epilepsy	Prospective	KD (*n* =1)	3mo: 100.0% (1/1)	-	-
Hosain et al. 2005 [52]	Infant epilepsy	Prospective	KD (*n* = 4)	3mo: 100.0% (4/4)	-	-
Kang et al. 2005 [53]	Infant epilepsy	Retrospective	KD (*n* = 49)	3mo: 57.1% (28/49)	-	3mo: 32.7% (16/49)
Coppola et al. 2002 [54]	Infant epilepsy	Prospective	KD (*n* = 1)	3mo: 100.0% (1/1)	-	3mo: 100.0% (1/1)
Takeoka et al. 2002 [55]	Infant epilepsy	Retrospective	KD (*n* = 1)	3mo: 100.0% (1/1)	-	3mo: 100.0% (1/1)
Maydell et al. 2001 [56]	Infant epilepsy	Retrospective	KD (*n* = 15)	3mo: 46.7% (7/15)	-	-
Nordli et al. 2001 [57]	Infant epilepsy	Retrospective	KD (*n* = 32)	3mo: 53.1% (17/32)	-	3mo: 18.8% (6/32)
Freeman et al. 1998 [58]	Infant epilepsy	Prospective	KD (*n* = 27)	3mo: 63.0% (17/27)	-	3mo: 0.0% (0/27)
Eun et al. 2006 [50]	Infantile spasms	Retrospective	KD (*n* = 43)	3mo: 69.8% (30/43)<br>6mo: 53.5% (23/43)	-	3mo: 34.9% (15/43)<br>6mo: 39.5% (17/43)
Kossoff et al. 2002 [59]	Infantile spasms	Retrospective	KD (*n* = 23)	3mo: 60.9% (14/23)<br>6mo: 56.5% (13/23)<br>12mo: 56.5% (13/23)	3mo: 11.3% (8/71)<br>6mo: 9.9% (7/71)<br>12mo: 8.5% (6/71)	3mo: 4.2% (3/71)<br>6mo: 4.2% (3/71)<br>12mo: 4.2% (3/71)
Caraballo et al. 2020 [60]	Lennox Gastaut syndrome	Retrospective	KD (*n* = 18)	18mo: 44.4% (8/18)	-	18mo: 16.7% (3/18)
Zhang et al. 2016 [61]	Lennox-Gastaut syndrome	Retrospective	KD (*n* = 47)	3mo: 48.9% (23/47)<br>6mo: 44.7% (21/47)	6mo: 19.1% (9/47)	6mo: 8.5% (4/47)
Lemmon et al. 2012 [62]	Lennox-Gastaut syndrome	Retrospective	KD (*n* = 71)	3mo: 74.6% (53/71)<br>6mo: 50.7% (36/71)<br>12mo: 43.7% (31/71)	3mo: 28.2% (20/71)<br>6mo: 22.5% (16/71)<br>12mo: 19.7% (14/71)	3mo: 4.2% (3/71)<br>6mo: 1.4% (1/71)<br>12mo: 1.4% (1/71)
Caraballo et al. 2011 [63]	Lennox Gastaut syndrome	Retrospective	KD (*n* = 19)	18mo: 63.2% (12/19)	-	18mo: 36.8% (7/19)
Lambrechts et al. 2017 [64]	Pediatric epilepsy	RCT	KD (*n* = 26)	4mo: 50.0% (13/26)	-	-
El-Rashidy et al.2013 [65]	Pediatric epilepsy	RCT	KD (*n* = 10)	6mo: 100.0% (10/10)	-	-
Neal et al. 2008 [66]	Pediatric epilepsy	RCT	KD (*n* = 54)	3mo: 38.9% (21/54)	-	-

Lambrechts et al. 2012 [67]	Refractory adult epilepsy	Prospective	KD (*n* = 9)	4mo: 11.1% (1/9)<br>6mo: 16.7% (1/6)<br>12mo: 40.0% (2/5)		
Sirven et al. 1999 [68]	Refractory adult epilepsy	Prospective	KD (*n* = 11)	8mo: 54.5% (6/11)	8mo: 27.3% (3/11)	8mo: 9.1% (1/11)
Wu et al. 2016 [35]	Refractory childhood epilepsy	Retrospective	KD (*n* = 87)	3mo: 50.6% (44/87)<br>6mo: 51.7% (45/87)	3mo: 35.6% (31/87)<br>6mo: 37.9% (33/87)	3mo: 29.9% (26/87)<br>6mo: 32.2% (28/87)
Zamani et al. 2016 [69]	Refractory childhood epilepsy	Prospective	KD (*n* = 33)	3mo: 63.6% (21/33)<br>6mo: 63.6% (21/33)	-	3mo: 6.1% (2/33)
Zhu et al. 2016 [70]	Refractory childhood epilepsy	Prospective	KD (*n* = 42)	3mo: 69.0% (29/42)<br>6mo: 54.8% (23/42)<br>12mo: 40.5% (17/42)	-	3mo: 21.4% (9/42)<br>6mo: 26.2% (11/42)<br>12mo: 26.2% (11/42)
Hallböök et al. 2015 [71]	Refractory childhood epilepsy	Retrospective	KD (*n* = 315)	3mo: 50.5% (159/315)<br>6mo: 46.0% (145/315)<br>12mo: 41.9% (132/315)	-	3mo: 12.1% (38/315)<br>6mo: 14.9% (47/315)<br>12mo: 11.7% (37/315)
Kim et al. 2015 [34]	Refractory childhood epilepsy	RCT	KD (*n* = 51)	3mo: 43.1% (22/51)<br>6mo: 39.2% (20/51)	3mo: 37.3% (19/51)<br>6mo: 37.3% (19/51)	3mo: 33.3% (17/51)<br>6mo: 31.4% (16/51)
Bansal et al. 2014 [72]	Refractory childhood epilepsy	Prospective	KD (*n* = 60)	3mo: 50.0% (30/60)	3mo: 16.7% (10/60)	-
Kapetanakis et al. 2014 [73]	Refractory childhood epilepsy	Retrospective	KD (*n* = 43)	12mo: 27.9% (12/43)	-	12mo: 9.3% (4/43)
Kayyali et al. 2014 [74]	Refractory childhood epilepsy	Prospective	KD (*n* = 20)	3mo: 70.0% (14/20)<br>6mo: 65.0% (13/20)<br>12mo: 65.0% (13/20)	3mo: 20.0% (4/20)<br>6mo: 20.0% (4/20)<br>12mo: 30.0% (6/20)	-
Shull et al. 2014 [75]	Refractory childhood epilepsy	Retrospective	KD (*n* = 48)	-	-	-
Li et al. 2013 [76]	Refractory childhood epilepsy	Prospective	KD (*n* = 31)	3mo: 71.0% (22/31)	-	3mo: 45.2% (14/31)
Pires et al. 2013 [41]	Refractory childhood epilepsy	Prospective	KD (*n* = 17)	3mo: 88.2% (15/17)<br>6mo: 82.4% (14/17)	-	3mo: 64.7% (11/17)<br>6mo: 52.9% (9/17)

Suo et al. 2013 [77]	Refractory childhood epilepsy	Prospective	KD (*n* = 317)	3mo: 35.0% (111/317)<br>6mo: 26.2% (83/317)<br>12mo: 18.6% (59/317)	3mo: 23.7% (75/317)<br>6mo: 17.0% (54/317)<br>12mo: 13.9% (44/317)	3mo: 20.8% (66/317)<br>6mo: 13.6% (43/317)<br>12mo: 10.7% (34/317)
Dahlin et al. 2012 [78]	Refractory childhood epilepsy	Retrospective	KD (*n* = 26)	3mo: 65.4% (17/26)	3mo: 26.9% (7/26)	3mo: 19.2% (5/26)
Martins et al. 2012 [79]	Refractory childhood epilepsy	Retrospective	KD (*n* = 29)	6mo: 48.3% (14/29)<br>12mo: 27.6% (8/29)	-	6mo: 24.1% (7/29)<br>12mo: 13.8% (4/29)
Miranda et al. 2011 [80]	Refractory childhood epilepsy	Retrospective	KD (*n* = 50)	6mo: 60.0% (30/50)	6mo: 34.0% (17/50)	-
Raju et al. 2011 [81]	Refractory childhood epilepsy	RCT	KD (*n* = 19)	3mo: 57.9% (11/19)	-	3mo: 26.3% (5/19)
Beniczky et al. 2010 [82]	Refractory childhood epilepsy	Retrospective	KD (*n* = 50)	3mo: 66.0% (33/50)	3mo: 36.0% (18/50)	3mo: 18.0% (9/50)
Coppola et al. 2010 [46]	Refractory childhood epilepsy	Prospective	KD (*n* = 38)	3mo: 71.1% (27/38)<br>6mo: 73.7% (28/38)<br>12mo: 52.6% (20/38)	-	3mo: 28.9% (11/38)<br>6mo: 31.6% (12/38)<br>12mo: 23.7% (9/38)
Hong et al. 2010 [83]	Refractory childhood epilepsy	Prospective	KD (*n* = 104)	3mo: 63.5% (66/104)<br>6mo: 64.4% (67/104)<br>12mo: 76.9% (80/104)	3mo: 31.7% (33/104)<br>6mo: 39.4% (41/104)<br>12mo: 43.3% (45/104)	3mo: 18.3% (19/104)<br>6mo: 27.9% (29/104)<br>12mo: 29.8% (31/104)
Lee et al. 2010 [84]	Refractory childhood epilepsy	Prospective	KD (*n* = 28)	-	3mo: 57.1% (16/28)	3mo: 32.1% (9/28)
Mirjavadi et al. 2010 [85]	Refractory childhood epilepsy	Retrospective	KD (*n* = 66)	3mo: 59.1% (39/66)	-	3mo: 18.2% (12/66)
Neal et al. 2009 [86]	Refractory childhood epilepsy	RCT	KD (*n* = 73)	3mo: 24.7% (18/73)<br>6mo: 24.7% (18/73)<br>12mo: 17.8% (13/73)	3mo: 6.8% (5/73)<br>6mo: 8.2% (6/73)<br>12mo: 9.6% (7/73)	3mo: 1.4% (1/73)<br>6mo: 1.4% (1/73)<br>12mo: 5.5% (4/73)
Porta et al. 2009 [87]	Refractory childhood epilepsy	Retrospective	KD (*n* = 17)	3mo: 64.7% (11/17)<br>6mo: 41.2% (7/17)<br>12mo: 23.5% (4/17)	3mo: 41.2% (7/17)<br>6mo: 23.5% (4/17)<br>12mo: 11.8% (2/17)	-
Sharma et al. 2009 [88]	Refractory childhood epilepsy	Prospective	KD (*n* = 27)	3mo: 59.3% (16/27)<br>6mo: 48.1% (13/27)<br>12mo: 37.0% (10/27)	3mo: 25.9% (7/27)<br>6mo: 22.2% (6/27)<br>12mo: 25.9% (7/27)	3mo: 11.1% (3/27)<br>6mo: 14.8% (4/27)<br>12mo: 18.5% (5/27)

Spulber et al. 2009 [89]	Refractory childhood epilepsy	Prospective	KD (*n* = 22)	12mo: 63.6% (14/22)	12mo: 31.8% (7/22)	12mo: 27.3% (6/22)
Villeneuve et al 2009 [48]	Refractory childhood epilepsy	Retrospective	KD (*n* = 22)	-	-	-
Jung et al. 2008 [90]	Refractory childhood epilepsy	Retrospective	KD (*n* = 47)	3mo: 61.7% (29/47)	-	3mo: 44.7% (21/47)
Kossoff et al. 2008 [91]	Refractory childhood epilepsy	Retrospective	KD (*n* = 118)	3mo: 75.4% (89/118)<br>6mo: 71.2% (84/118)	3mo: 48.3% (57/118)<br>6mo: 43.2% (51/118)	-
Remahl et al. 2008 [92]	Refractory childhood epilepsy	Prospective	KD (*n* = 23)	3mo: 69.6% (16/23)	3mo: 17.4% (4/23)	3mo: 13.0% (3/23)
Stainman et al. 2007 [93]	Refractory childhood epilepsy	Retrospective	KD (*n* = 45)	6mo: 75.6% (34/45)	6mo: 60.0% (27/45)	6mo: 28.9% (13/45)
Freitas et al. 2007 [94]	Refractory childhood epilepsy	Retrospective	KD (*n* = 70)	6mo: 62.9% (44/70)<br>12mo: 54.3% (38/70)	-	-
Dahlin et al. 2007 [49]	Refractory childhood epilepsy	Prospective	KD (*n* = 25)	3mo: 64.0% (16/25)<br>6mo: 60.0% (15/25)<br>12mo: 48.0% (12/25)	3mo: 16.0% (4/25)<br>6mo: 32.0% (8/25)<br>12mo: 24.0% (6/25)	3mo: 8.0% (2/25)<br>6mo: 12.0% (3/25)<br>12mo: 8.0% (2/25)
Hallböök et al. 2007 [95]	Refractory childhood epilepsy	Prospective	KD (*n* = 18)	3mo: 66.7% (12/18)<br>12mo: 44.4% (8/18)	3mo: 44.4% (8/18)<br>12mo: 22.2% (4/18)	3mo: 22.2% (4/18)<br>12mo: 11.1% (2/18)
Seo et al. 2007 [96]	Refractory childhood epilepsy	RCT	KD (*n* = 36)	3mo: 72.2% (26/36)	3mo: 36.1% (13/36)	3mo: 30.6% (11/36)
Seo et al. 2007 [96]	Refractory childhood epilepsy	RCT	KD (*n* = 40)	3mo: 85.0% (34/40)	3mo: 60.0% (24/40)	3mo: 55.0% (22/40)
Rizzutti et al. 2007 [97]	Refractory childhood epilepsy	Prospective	KD (*n* = 46)	12mo: 67.4% (31/46)	12mo: 32.6% (15/46)	12mo: 17.4% (8/46)
Kang et al. 2005 [53]	Refractory childhood epilepsy	Retrospective	KD (*n* = 199)	3mo: 61.8% (123/199)<br>6mo: 57.8% (115/199)<br>12mo: 41.2% (82/199)	-	3mo: 35.2% (70/199)<br>6mo: 33.2% (66/199)<br>12mo: 25.1% (50/199)
Kossoff et al. 2005 [98]	Refractory childhood epilepsy	Retrospective	KD (*n* = 12)	6mo: 91.7% (11/12)	6mo: 66.7% (8/12)	6mo: 25.0% (3/12)
Mackay et al. 2005 [99]	Refractory childhood epilepsy	Retrospective	KD (*n* = 26)	3mo: 26.9% (7/26)<br>6mo: 19.2% (5/26)<br>12mo: 30.8% (8/26)	-	-

Lyczkowski et al. 2005 [100]	Refractory childhood epilepsy	Retrospective	KD (n = 71)	3mo: 62.0% (44/71)<br>6mo: 46.5% (33/71)<br>12mo: 32.4% (23/71)	3mo: 32.4% (23/71)<br>6mo: 28.2% (20/71)<br>12mo: 21.1% (15/71)	-
Peterson et al. 2005 [101]	Refractory childhood epilepsy	Retrospective	KD (n = 57)	12mo: 54.4% (31/57)	12mo: 33.3% (19/57)	-
Hosain et al. 2004 [52]	Refractory childhood epilepsy	Prospective	KD (n = 12)	12mo: 83.3% (10/12)	12mo: 50.0% (6/12)	12mo: 0.0% (0/12)
Kim et al. 2004 [102]	Refractory childhood epilepsy	Retrospective	KD (n = 83)	3mo: 75.9% (63/83)	3mo: 53.0% (44/83)	3mo: 34.9% (29/83)
Kim et al. 2004 [102]	Refractory childhood epilepsy	Prospective	KD (n = 41)	3mo: 75.6% (31/41)	3mo: 53.7% (22/41)	3mo: 34.1% (14/41)
DiMario et al. 2002 [103]	Refractory childhood epilepsy	Retrospective	KD (n = 48)	6mo: 35.4% (17/48)<br>12mo: 22.9% (11/48)	-	6mo: 8.3% (4/48)<br>12mo: 8.3% (4/48)
Coppola et al. 2001 [54]	Refractory childhood epilepsy	Prospective	KD (n = 56)	3mo: 37.5% (21/56)<br>6mo: 26.8% (15/56)<br>12mo: 8.9% (5/56)	-	3mo: 10.7% (6/56)<br>6mo: 7.1% (4/56)<br>12mo: 0.0% (0/56)
Kankirawatana 2001 [104]	Refractory childhood epilepsy	Prospective	KD (n = 35)	3mo: 48.6% (17/35)<br>6mo: 42.9% (15/35)<br>12mo: 28.6% (10/35)	3mo: 42.9% (15/35)<br>6mo: 34.3% (12/35)<br>12mo: 22.9% (8/35)	3mo: 14.3% (5/35)<br>6mo: 11.4% (4/35)<br>12mo: 8.6% (3/35)
Vining 1998 [105]	Refractory childhood epilepsy	Prospective	KD (n = 51)	3mo: 54.9% (28/51)<br>6mo: 52.9% (27/51)<br>12mo: 39.2% (20/51)	3mo: 25.5% (13/51)<br>6mo: 29.4% (15/51)<br>12mo: 21.6% (11/51)	3mo: 11.8% (6/51)<br>12mo: 9.8% (5/51)
Freeman 1998 [58]	Refractory childhood epilepsy	Prospective	KD (n = 150)	3mo: 59.3% (89/150)<br>6mo: 51.3% (77/150)<br>12mo: 50.0% (75/150)	3mo: 33.3% (50/150)<br>6mo: 32.0% (48/150)<br>12mo: 27.3% (41/150)	3mo: 2.7% (4/150)<br>6mo: 3.3% (5/150)<br>12mo: 7.3% (11/150)
Maydell et al. 2001 [56]	Refractory epilepsy	Prospective	KD (n = 143)	3mo: 41.3% (59/143)<br>6mo: 42.0% (60/143)<br>12mo: 37.8% (54/143)	3mo: 30.1% (43/143)<br>6mo: 28.7% (41/143)<br>12mo: 26.6% (38/143)	3mo: 14.7% (21/143)<br>6mo: 16.8% (24/143)<br>12mo: 16.1% (23/143)
Mady et al. 2003 [106]	Refractory epilepsy in adolescents	Retrospective	KD (n = 45)	6mo: 31.1% (14/45)<br>12mo: 28.9% (13/45)	6mo: 17.8% (8/45)<br>12mo: 13.3% (6/45)	-

MAD						
Sharma et al. 2014 [107]	Lennox Gastaut syndrome	Retrospective	MAD (*n* = 25)	3mo: 48.0% (12/25)<br>6mo: 44.0% (11/25)<br>12mo: 36.0% (9/25)	3mo: 24.0% (6/25)<br>6mo: 28.0% (7/25)<br>12mo: 32.0% (8/25)	3mo: 8.0% (2/25)<br>6mo: 12.0% (3/25)<br>12mo: 12.0% (3/25)
El-Rashidy et al.2013 [65]	Pediatric epilepsy	RCT	MAD (*n* = 15)	6mo: 40.0% (6/15)	-	-
Cervenka et al. 2016 [108]	Refractory adult epilepsy	Prospective	MAD (*n* = 106)	3mo: 35.8% (38/106)<br>6mo: 30.5% (32/105)<br>12mo: 29.6% (29/98)	-	3mo: 16.0% (17/106)<br>6mo: 14.3% (15/105)<br>12mo: 13.3% (13/98)
Vaccarezza et al. 2014 [109]	Refractory adult epilepsy	Prospective	MAD (*n* = 3)	3mo: 33.3% (1/3)	-	-
Kossoff et al. 2013 [110]	Refractory adult epilepsy	Prospective	MAD (*n* = 8)	3mo: 62.5% (5/8)	-	3mo: 25.0% (2/8)
Cervenka et al. 2012 [111]	Refractory adult epilepsy	Prospective	MAD (*n* = 22)	3mo: 27.3% (6/22)	3mo: 13.6% (3/22)	3mo: 4.5% (1/22)
Smith et al. 2011 [112]	Refractory adult epilepsy	Prospective	MAD (*n* = 17)	3mo: 11.8% (2/17)<br>6mo: 28.6% (4/14)<br>12mo: 21.4% (3/14)	-	-
Kossoff et al. 2008 [113]	Refractory adult epilepsy	Prospective	MAD (*n* = 18)	3mo: 72.2% (13/18)<br>6mo: 50.0% (9/18)	-	3mo: 5.6% (1/18)<br>6mo: 5.6% (1/18)
Sharma et al. 2016 [114]	Refractory childhood epilepsy	RCT	MAD (*n* = 41)	3mo: 56.1% (23/41)	3mo: 19.5% (8/41)	3mo: 14.6% (6/41)
Kim et al. 2015 [34]	Refractory childhood epilepsy	RCT	MAD (*n* = 53)	3mo: 41.5% (22/53)<br>6mo: 35.8% (19/53)	3mo: 32.1% (17/53)<br>6mo: 30.2% (16/53)	3mo: 24.5% (13/53)<br>6mo: 22.6% (12/53)
Vaccarezzaa 2014 [115]	Refractory childhood epilepsy	Retrospective	MAD (*n* = 9)	6mo: 66.7% (6/9)	6mo: 22.2% (2/9)	-
Sharma et al. 2013 [116]	Refractory childhood epilepsy	RCT	MAD (*n* = 50)	3mo: 52.0% (26/50)	3mo: 30.0% (15/50)	3mo: 10.0% (5/50)
Chen et al. 2012 [117]	Refractory childhood epilepsy	Retrospective	MAD (*n* = 87)	6mo: 41.4% (36/87)<br>12mo: 37.9% (33/87)	6mo: 27.6% (24/87)<br>12mo: 28.7% (25/87)	12mo: 18.4% (16/87)
Sharma et al. 2012 [45]	Refractory childhood epilepsy	Prospective	MAD (*n* = 15)	-	6mo: 60.0% (9/15)	3mo: 40.0% (6/15)

						6mo: 40.0% (6/15)
Miranda et al. 2011 [80]	Refractory childhood epilepsy	Prospective	MAD (n = 33)	3mo: 51.5% (17/33)<br>6mo: 39.4% (13/33)<br>12mo: 27.3% (9/33)	3mo: 42.4% (14/33)<br>6mo: 18.2% (6/33)<br>12mo: 12.1% (4/33)	3mo: 15.2% (5/33)<br>6mo: 0.0% (0/33)<br>12mo: 0.0% (0/33)
Tonekaboni et al. 2010 [47]	Refractory childhood epilepsy	Prospective	MAD (n = 51)	3mo: 31.4% (16/51)	3mo: 11.8% (6/51)	3mo: 9.8% (5/51)
Barzegar et al. 2010 [118]	Refractory childhood epilepsy	Prospective	MAD (n = 21)	3mo: 57.1% (12/21)<br>6mo: 42.9% (9/21)	3mo: 33.3% (7/21)<br>6mo: 33.3% (7/21)	-
Porta et al. 2009 [87]	Refractory childhood epilepsy	Retrospective	MAD (n = 10)	3mo: 20.0% (2/10)<br>6mo: 20.0% (2/10)<br>12mo: 10.0% (1/10)	3mo: 10.0% (1/10)<br>6mo: 20.0% (2/10)<br>12mo: 10.0% (1/10)	-
Kang et al. 2007 [119]	Refractory childhood epilepsy	Prospective	MAD (n = 14)	3mo: 50.0% (7/14)<br>6mo: 35.7% (5/14)	-	3mo: 28.6% (4/14)<br>6mo: 21.4% (3/14)
Kossoff et al. 2006 [120]	Refractory childhood epilepsy	Prospective	MAD (n = 20)	3mo: 70.0% (14/20)<br>6mo: 65.0% (13/20)	3mo: 25.0% (5/20)<br>6mo: 35.0% (7/20)	3mo: 15.0% (3/20)<br>6mo: 20.0% (4/20)
Weber et al. 2009 [121]	Refractory epilepsy in children and adolescents	Prospective	MAD (n = 15)	3mo: 40.0% (6/15)	3mo: 13.3% (2/15)	-
Kossoff et al. 2010 [122]	Sturge-Weber syndrome	Prospective	MAD (n = 5)	3mo: 60.0% (3/5)<br>6mo: 60.0% (3/5)	3mo: 40.0% (2/5)<br>6mo: 40.0% (2/5)	3mo: 20.0% (1/5)<br>6mo: 20.0% (1/5)
Control group						
Stainman et al. 2007 [93]	Childhood epilepsy	Retrospective	Surgically approachable(n = 45)	6mo: 62.2% (28/45)	6mo: 26.7% (12/45)	6mo: 13.3% (6/45)
Dressler et al. 2019 [28]	Infant epilepsy	Prospective	Adrenocorticotropic hormone(n = 16)	3mo: 68.8% (11/16)	-	3mo: 43.8% (7/16)
El-Rashidy et al.2013 [65]	Pediatric epilepsy	RCT	Usual care(n = 15)	6mo: 6.6% (1/15)	-	-
Neal et al. 2008 [66]	Pediatric epilepsy	RCT	Usual care(n = 49)	3mo: 6.1% (3/49)	-	-

Lambrechts et al. 2017 [64]	Pediatric epilepsy	RCT	Usual care(n = 22)	4mo: 15.4% (4/26)	-	-
Sharma et al. 2016 [114]	Refractory childhood epilepsy	RCT	Routine diet(n = 40)	3mo: 7.5% (3/40)	3mo: 5.0% (2/40)	3mo: 5.0% (2/40)
Sharma et al. 2013 [116]	Refractory childhood epilepsy	RCT	Control group(n = 52)	3mo: 11.5% (6/52)	3mo: 13.5% (7/52)	3mo: 0.0% (0/52)

Abbreviation. CON, control; KD, Ketogenic Diet; MAD, Modified Atkins diet; RCT, randomized controlled trial; ≥50%, proportion ≥50% seizure reduction; ≥90%, proportion ≥90% seizure reduction; SF, seizure freedom; N/A, not available; SF, seizure free; RCT, randomized controlled trial; mo, month.

Reference:

1. Castellana, M., et al., *Efficacy and safety of very low calorie ketogenic diet (VLCKD) in patients with overweight and obesity: A systematic review and meta-analysis.* Rev Endocr Metab Disord, 2020. **21**(1): p. 5-16.
2. Choi, Y.J., S.M. Jeon, and S. Shin, *Impact of a Ketogenic Diet on Metabolic Parameters in Patients with Obesity or Overweight and with or without Type 2 Diabetes: A Meta-Analysis of Randomized Controlled Trials.* Nutrients, 2020. **12**(7).
3. Muscogiuri, G., et al., *European Guidelines for Obesity Management in Adults with a Very Low-Calorie Ketogenic Diet: A Systematic Review and Meta-Analysis.* Obes Facts, 2021. **14**(2): p. 222-245.
4. Cao, J., et al., *The Effect of a Ketogenic Low-Carbohydrate, High-Fat Diet on Aerobic Capacity and Exercise Performance in Endurance Athletes: A Systematic Review and Meta-Analysis.* Nutrients, 2021. **13**(8).
5. Bueno, N.B., et al., *Very-low-carbohydrate ketogenic diet v. low-fat diet for long-term weight loss: a meta-analysis of randomised controlled trials.* Br J Nutr, 2013. **110**(7): p. 1178-87.
6. Gibson, A.A., et al., *Do ketogenic diets really suppress appetite? A systematic review and meta-analysis.* Obes Rev, 2015. **16**(1): p. 64-76.
7. Sourbron, J., et al., *Ketogenic diet for the treatment of pediatric epilepsy: review and meta-analysis.* Childs Nerv Syst, 2020. **36**(6): p. 1099-1109.
8. Yang, Y.F., et al., *Efficacy of Low-Carbohydrate Ketogenic Diet as an Adjuvant Cancer Therapy: A Systematic Review and Meta-Analysis of Randomized Controlled Trials.* Nutrients, 2021. **13**(5).
9. Whittaker, J. and M. Harris, *Low-carbohydrate diets and men's cortisol and testosterone: Systematic review and meta-analysis.* Nutr Health, 2022. **28**(4): p. 543-554.
10. Martin-McGill, K.J., et al., *Ketogenic diets for drug-resistant epilepsy.* Cochrane Database Syst Rev, 2020. **6**(6): p. Cd001903.
11. Zhou, C., et al., *Ketogenic Diet Benefits to Weight Loss, Glycemic Control, and Lipid Profiles in Overweight Patients with Type 2 Diabetes Mellitus: A Meta-Analysis of Randomized Controlled Trails.* Int J Environ Res Public Health, 2022. **19**(16).
12. Pizzo, F., et al., *Ketogenic diet in pediatric seizures: a randomized controlled trial review and meta-analysis.* Expert Rev Neurother, 2022. **22**(2): p. 169-177.
13. Amini, M.R., et al., *The effect of ketogenic diet on body composition and anthropometric measures: A systematic review and meta-analysis of randomized controlled trials.* Crit Rev Food Sci Nutr, 2022. **62**(13): p. 3644-3657.
14. Taftian, M., et al., *The Effect of Ketogenic Diet on Weight Loss in Adult Patients with Cancer: A Systematic Review and Meta-Analysis of Controlled Clinical Trials.* Nutr Cancer, 2022. **74**(4): p. 1222-1234.

15. Zhang, J., et al., *Efficacy of ketogenic diet in CDKL5-related epilepsy: a single arm meta-analysis.* Orphanet J Rare Dis, 2022. **17**(1): p. 385.
16. Lee, H.S. and J. Lee, *Effects of Combined Exercise and Low Carbohydrate Ketogenic Diet Interventions on Waist Circumference and Triglycerides in Overweight and Obese Individuals: A Systematic Review and Meta-Analysis.* Int J Environ Res Public Health, 2021. **18**(2).
17. Lee, H.S. and J. Lee, *Influences of Ketogenic Diet on Body Fat Percentage, Respiratory Exchange Rate, and Total Cholesterol in Athletes: A Systematic Review and Meta-Analysis.* Int J Environ Res Public Health, 2021. **18**(6).
18. Ashtary-Larky, D., et al., *Effects of resistance training combined with a ketogenic diet on body composition: a systematic review and meta-analysis.* Crit Rev Food Sci Nutr, 2022. **62**(21): p. 5717-5732.
19. Liu, F.Y., et al., *Efficacy of the ketogenic diet in Chinese children with Dravet syndrome: A focus on neuropsychological development.* Epilepsy & Behavior, 2019. **92**: p. 98-102.
20. Liu, J., et al., *Prospective control study of efficacy and safety of ketogenic diet on drug refractory Dravet syndrome.* Chinese Journal of Applied Clinical Pediatrics, 2019. **34**(15): p. 1160-1165.
21. Ni, Y., et al., *Prospective study of the efficacy of a ketogenic diet in 20 patients with Dravet syndrome.* Seizure-European Journal of Epilepsy, 2018. **60**: p. 144-148.
22. Tian, X.J., et al., *The Efficacy of Ketogenic Diet in 60 Chinese Patients With Dravet Syndrome.* Frontiers in Neurology, 2019. **10**.
23. Dressler, A., et al., *Efficacy and tolerability of the ketogenic diet in Dravet syndrome - Comparison with various standard antiepileptic drug regimen.* Epilepsy Research, 2015. **109**: p. 81-89.
24. Caraballo, R.H., *Nonpharmacologic treatments of Dravet syndrome: Focus on the ketogenic diet.* Epilepsia, 2011. **52**: p. 79-82.
25. Nabbout, R., et al., *Ketogenic diet also benefits Dravet syndrome patients receiving stiripentol: A prospective pilot study.* Epilepsia, 2011. **52**(7): p. E54-E57.
26. Caraballo, R.H., et al., *Ketogenic diet in patients with Dravet syndrome.* Epilepsia, 2005. **46**(9): p. 1539-1544.
27. Nei, M., et al., *Ketogenic diet in adolescents and adults with epilepsy.* Seizure, 2014. **23**(6): p. 439-42.
28. Dressler, A., et al., *Efficacy and tolerability of the ketogenic diet versus high-dose adrenocorticotropic hormone for infantile spasms: A single-center parallel-cohort randomized controlled trial.* Epilepsia, 2019. **60**(3): p. 441-451.
29. Dressler, A., et al., *Ketogenic parenteral nutrition in 17 pediatric patients with epilepsy.* Epilepsia open, 2018. **3**(1): p. 30-39.
30. Ismayilova, N., et al., *Ketogenic diet therapy in infants less than two years of age for medically refractory epilepsy.* Seizure-European Journal of Epilepsy, 2018. **57**: p. 5-7.

31. Wirrell, E., et al., *Ketogenic Diet Therapy in Infants: Efficacy and Tolerability.* Pediatric Neurology, 2018. **82**: p. 13-18.
32. Zhang, Y.J., et al., *Altered gut microbiome composition in children with refractory epilepsy after ketogenic diet.* Epilepsy Research, 2018. **145**: p. 163-168.
33. Sampaio, L.P.D., C. Takakura, and M.L.G. de Manreza, *The use of a formula-based ketogenic diet in children with refractory epilepsy.* Arquivos De Neuro-Psiquiatria, 2017. **75**(4): p. 234-237.
34. Kim, J.A., et al., *Efficacy of the classic ketogenic and the modified Atkins diets in refractory childhood epilepsy.* Epilepsia, 2016. **57**(1): p. 51-58.
35. Wu, Y.J., et al., *Six-month efficacy of the Ketogenic diet is predicted after 3 months and is unrelated to clinical variables.* Epilepsy & Behavior, 2016. **55**: p. 165-169.
36. Dressler, A., et al., *The ketogenic diet in infants - Advantages of early use.* Epilepsy Research, 2015. **116**: p. 53-58.
37. Hirano, Y., et al., *Ketogenic diet therapy can improve ACTH-resistant West syndrome in Japan.* Brain & Development, 2015. **37**(1): p. 18-22.
38. van der Louw, E., et al., *Concomitant lamotrigine use is associated with decreased efficacy of the ketogenic diet in childhood refractory epilepsy.* Seizure-European Journal of Epilepsy, 2015. **32**: p. 75-77.
39. Vehmeijer, F.O.L., et al., *Can we predict efficacy of the ketogenic diet in children with refractory epilepsy?* European Journal of Paediatric Neurology, 2015. **19**(6): p. 701-705.
40. Caraballo, R.H., et al., *Ketogenic diet in pediatric patients with refractory focal status epilepticus.* Epilepsy Research, 2014. **108**(10): p. 1912-1916.
41. Pires, M.E., et al., *Ketogenic diet for infantile spasms refractory to first-line treatments: An open prospective study.* Epilepsy Research, 2013. **105**(1-2): p. 189-194.
42. Jung, D.E., et al., *Safety and role of ketogenic parenteral nutrition for intractable childhood epilepsy.* Brain & Development, 2012. **34**(8): p. 620-624.
43. Kumada, T., et al., *Efficacy and tolerability of modified Atkins diet in Japanese children with medication-resistant epilepsy.* Brain & Development, 2012. **34**(1): p. 32-38.
44. Larson, A.M., H.H. Pfeifer, and E.A. Thiele, *Low glycemic index treatment for epilepsy in tuberous sclerosis complex.* Epilepsy Research, 2012. **99**(1-2): p. 180-182.
45. Sharma, S., et al., *Use of the modified Atkins diet in infantile spasms refractory to first-line treatment.* Seizure-European Journal of Epilepsy, 2012. **21**(1): p. 45-48.
46. Coppola, G., et al., *Ketogenic diet for the treatment of catastrophic epileptic encephalopathies in childhood.* European Journal of Paediatric Neurology, 2010. **14**(3): p. 229-234.

47. Tonekaboni, S.H., et al., *Efficacy of the Atkins Diet as Therapy for Intractable Epilepsy in Children.* Archives of Iranian Medicine, 2010. **13**(6): p. 492-497.
48. Villeneuve, N., et al., *The ketogenic diet improves recently worsened focal epilepsy.* Developmental Medicine and Child Neurology, 2009. **51**(4): p. 276-281.
49. Dahlin, M., et al., *Plasma phospholipid fatty acids are influenced by a ketogenic diet enriched with n-3 fatty acids in children with epilepsy.* Epilepsy Research, 2007. **73**(2): p. 199-207.
50. Eun, S.H., et al., *Ketogenic diet for treatment of infantile spasms.* Brain & Development, 2006. **28**(9): p. 566-571.
51. Dahlin, M., et al., *The ketogenic diet influences the levels of excitatory and inhibitory amino acids in the CSF in children with refractory epilepsy.* Epilepsy Research, 2005. **64**(3): p. 115-125.
52. Hosain, S.A., M. La Vega-Talbott, and G.E. Solomon, *Ketogenic diet in pediatric epilepsy patients with gastrostomy feeding.* Pediatric Neurology, 2005. **32**(2): p. 81-83.
53. Kang, H.C., et al., *Efficacy and safety of the ketogenic diet for intractable childhood epilepsy: Korean multicentric experience.* Epilepsia, 2005. **46**(2): p. 272-279.
54. Coppola, G., et al., *The ketogenic diet in children, adolescents and young adults with refractory epilepsy: an Italian multicentric experience.* Epilepsy Research, 2002. **48**(3): p. 221-227.
55. Takeoka, M., et al., *Concomitant treatment with topiramate and ketogenic diet in pediatric epilepsy.* Epilepsia, 2002. **43**(9): p. 1072-1075.
56. Maydell, B.V., et al., *Efficacy of the ketogenic diet in focal versus generalized seizures.* Pediatric Neurology, 2001. **25**(3): p. 208-212.
57. Nordli, D.R., et al., *Experience with the ketogenic diet in infants.* Pediatrics, 2001. **108**(1): p. 129-133.
58. Freeman, J.M., et al., *The efficacy of the ketogenic diet-1998: a prospective evaluation of intervention in 150 children.* Pediatrics, 1998. **102**(6): p. 1358-63.
59. Kossoff, E.H., et al., *Efficacy of the ketogenic diet for infantile spasms.* Pediatrics, 2002. **109**(5): p. 780-783.
60. Caraballo, R.H., et al., *Ketogenic diet in patients with Lennox-Gastaut syndrome.* Seizure-European Journal of Epilepsy, 2014. **23**(9): p. 751-755.
61. Zhang, Y.J., et al., *Therapeutic effects of the ketogenic diet in children with Lennox-Gastaut syndrome.* Epilepsy Research, 2016. **128**: p. 176-180.
62. Lemmon, M.E., et al., *Efficacy of the ketogenic diet in Lennox-Gastaut syndrome: a retrospective review of one institution's experience and summary of the literature.* Developmental Medicine and Child Neurology, 2012. **54**(5): p. 464-468.
63. Caraballo, R., et al., *Long-term follow-up of the ketogenic diet for refractory epilepsy: Multicenter Argentinean experience in 216 pediatric patients.* Seizure-European Journal of Epilepsy, 2011. **20**(8): p. 640-645.

64. Lambrechts, D., et al., *A randomized controlled trial of the ketogenic diet in refractory childhood epilepsy.* Acta Neurologica Scandinavica, 2017. **135**(2): p. 231-239.
65. El-Rashidy, O.F., et al., *Modified Atkins diet vs classic ketogenic formula in intractable epilepsy.* Acta Neurologica Scandinavica, 2013. **128**(6): p. 402-408.
66. Neal, E.G., et al., *The ketogenic diet for the treatment of childhood epilepsy: a randomised controlled trial.* Lancet Neurology, 2008. **7**(6): p. 500-506.
67. Lambrechts, D.A., et al., *The ketogenic diet as a treatment option in adults with chronic refractory epilepsy: efficacy and tolerability in clinical practice.* Epilepsy Behav, 2012. **23**(3): p. 310-4.
68. Sirven, J., et al., *The ketogenic diet for intractable epilepsy in adults: preliminary results.* Epilepsia, 1999. **40**(12): p. 1721-6.
69. Zamani, G.R., et al., *The effects of classic ketogenic diet on serum lipid profile in children with refractory seizures.* Acta Neurologica Belgica, 2016. **116**(4): p. 529-534.
70. Zhu, D.N., et al., *Ketogenic diet effects on neurobehavioral development of children with intractable epilepsy: A prospective study.* Epilepsy & Behavior, 2016. **55**: p. 87-91.
71. Hallbook, T., et al., *Effectiveness of the ketogenic diet used to treat resistant childhood epilepsy in Scandinavia.* European Journal of Paediatric Neurology, 2015. **19**(1): p. 29-36.
72. Bansal, S., et al., *The Ketogenic Diet: Initiation at Goal Calories Versus Gradual Caloric Advancement.* Pediatric Neurology, 2014. **50**(1): p. 26-30.
73. Kapetanakis, M., et al., *Effects of ketogenic diet on vascular function.* European Journal of Paediatric Neurology, 2014. **18**(4): p. 489-494.
74. Kayyali, H.R., et al., *Ketogenic Diet Efficacy in the Treatment of Intractable Epileptic Spasms.* Pediatric Neurology, 2014. **50**(3): p. 224-227.
75. Shull, S., et al., *Early Efficacy of the Ketogenic Diet Is Not Affected by Initial Body Mass Index Percentile.* Pediatric Neurology, 2014. **50**(5): p. 469-473.
76. Li, B.M., et al., *Effects of ketogenic diet on the clinical and electroencephalographic features of children with drug therapy-resistant epilepsy.* Experimental and Therapeutic Medicine, 2013. **5**(2): p. 611-615.
77. Suo, C.Q., et al., *Efficacy and safety of the ketogenic diet in Chinese children.* Seizure-European Journal of Epilepsy, 2013. **22**(3): p. 174-178.
78. Dahlin, M., J.E. Mansson, and P. Amark, *CSF levels of dopamine and serotonin, but not norepinephrine, metabolites are influenced by the ketogenic diet in children with epilepsy.* Epilepsy Research, 2012. **99**(1-2): p. 132-138.
79. Martins, L.D., et al., *Effect of the classic ketogenic diet on the treatment of refractory epileptic seizures.* Revista De Nutricao-Brazilian Journal of Nutrition, 2012. **25**(5): p. 565-573.
80. Miranda, M.J., et al., *Danish study of a Modified Atkins diet for medically intractable epilepsy in children: Can we achieve the same results as with the*

classical ketogenic diet? Seizure-European Journal of Epilepsy, 2011. **20**(2): p. 151-155.
81. Raju, K.N.V., et al., *Efficacy of 4:1 (classic) versus 2.5:1 ketogenic ratio diets in refractory epilepsy in young children: A randomized open labeled study.* Epilepsy Research, 2011. **96**(1-2): p. 96-100.
82. Beniczky, S., et al., *Effectiveness of the ketogenic diet in a broad range of seizure types and EEG features for severe childhood epilepsies.* Acta Neurologica Scandinavica, 2010. **121**(1): p. 58-62.
83. Hong, A.M., et al., *Infantile spasms treated with the ketogenic diet: Prospective single-center experience in 104 consecutive infants.* Epilepsia, 2010. **51**(8): p. 1403-1407.
84. Lee, Y.J., et al., *Usefulness of liquid ketogenic milk for intractable childhood epilepsy.* e-SPEN, the European e-Journal of Clinical Nutrition and Metabolism, 2010. **5**(5): p. e203-e207.
85. Alireza, M., et al., *Efficacy of the ketogenic diet as a therapy for intractable epilepsy in children.* Iranian Journal of Child Neurology, 2010. **4**.
86. Neal, E.G., et al., *A randomized trial of classical and medium-chain triglyceride ketogenic diets in the treatment of childhood epilepsy.* Epilepsia, 2009. **50**(5): p. 1109-1117.
87. Porta, N., et al., *Comparison of seizure reduction and serum fatty acid levels after receiving the ketogenic and modified Atkins diet.* Seizure-European Journal of Epilepsy, 2009. **18**(5): p. 359-364.
88. Sharma, S., et al., *Seizure control and biochemical profile on the ketogenic diet in young children with refractory epilepsy-Indian experience.* Seizure-European Journal of Epilepsy, 2009. **18**(6): p. 446-449.
89. Spulber, G., et al., *Growth dependence on insulin-like growth factor-1 during the ketogenic diet.* Epilepsia, 2009. **50**(2): p. 297-303.
90. Jung, D.E., H.C. Kang, and H.D. Kim, *Long-term outcome of the ketogenic diet for intractable childhood epilepsy with focal malformation of cortical development.* Pediatrics, 2008. **122**(2): p. E330-E333.
91. Kossoff, E.H., et al., *When do seizures usually improve with the ketogenic diet?* Epilepsia, 2008. **49**(2): p. 329-33.
92. Remahl, S., M.G. Dahlin, and P.E. Amark, *Influence of the ketogenic diet on 24-hour electroencephalogram in children with epilepsy.* Pediatric Neurology, 2008. **38**(1): p. 38-43.
93. Stainman, R.S., et al., *Decreased relative efficacy of the ketogenic diet for children with surgically approachable epilepsy.* Seizure-European Journal of Epilepsy, 2007. **16**(7): p. 615-619.
94. Freitas, A., et al., *Ketogenic diet for the treatment of refractory epilepsy - A 10 year experience in children.* Arquivos De Neuro-Psiquiatria, 2007. **65**(2B): p. 381-384.
95. Hallbook, T., et al., *Effects of ketogenic diet on epileptiform activity in children with therapy resistant epilepsy.* Epilepsy Research, 2007. **77**(2-3): p. 134-140.

96. Seo, J.H., et al., *Efficacy and tolerability of the ketogenic diet according to Lipid : Nonlipid ratios-comparison of 3 : 1 with 4 : 1 diet.* Epilepsia, 2007. **48**(4): p. 801-805.
97. Rizzutti, S., et al., *Is hospitalization really necessary during the introduction of the ketogenic diet?* Journal of Child Neurology, 2007. **22**(1): p. 33-37.
98. Kossoff, E.H., et al., *Tuberous sclerosis complex and the ketogenic diet.* Epilepsia, 2005. **46**(10): p. 1684-1686.
99. Mackay, M.T., et al., *The ketogenic diet in refractory childhood epilepsy.* Journal of Paediatrics and Child Health, 2005. **41**(7): p. 353-357.
100. Lyczkowski, D.A., et al., *Safety and tolerability of the ketogenic diet in pediatric epilepsy: Effects of valproate combination therapy.* Epilepsia, 2005. **46**(9): p. 1533-1538.
101. Peterson, S.J., et al., *Current research - Changes in growth and seizure reduction in children on the ketogenic diet as a treatment for intractable epilepsy.* Journal of the American Dietetic Association, 2005. **105**(5): p. 718-725.
102. Kim, D.W., et al., *Benefits of the nonfasting ketogenic diet compared with the initial fasting ketogenic diet.* Pediatrics, 2004. **114**(6): p. 1627-1630.
103. DiMario, F.J. and J. Holland, *The ketogenic diet: A review of the experience at Connecticut Children's Medical Center.* Pediatric Neurology, 2002. **26**(4): p. 288-292.
104. Kankirawatana, P., et al., *Ketogenic diet: an alternative treatment for refractory epilepsy in children.* Journal of the Medical Association of Thailand = Chotmaihet thangphaet, 2001. **84**(7): p. 1027-32.
105. Vining, E.P., et al., *A multicenter study of the efficacy of the ketogenic diet.* Archives of neurology, 1998. **55**(11): p. 1433-7.
106. Mady, M.A., et al., *The ketogenic diet: Adolescents can do it, too.* Epilepsia, 2003. **44**(6): p. 847-851.
107. Sharma, S., et al., *Use of the Modified Atkins Diet in Lennox Gastaut Syndrome.* Journal of Child Neurology, 2015. **30**(5): p. 576-579.
108. Cervenka, M.C., et al., *Establishing an Adult Epilepsy Diet Center: Experience, efficacy and challenges.* Epilepsy Behav, 2016. **58**: p. 61-8.
109. Vaccarezza, M.M., et al., *Treatment of refractory epilepsy with the modified Atkins diet.* Arch Argent Pediatr, 2014. **112**(4): p. 348-51.
110. Kossoff, E.H., B.J. Henry, and M.C. Cervenka, *Efficacy of dietary therapy for juvenile myoclonic epilepsy.* Epilepsy Behav, 2013. **26**(2): p. 162-4.
111. Cervenka, M.C., et al., *E-mail management of the modified Atkins Diet for adults with epilepsy is feasible and effective.* Epilepsia, 2012. **53**(4): p. 728-32.
112. Smith, M., et al., *Efficacy and tolerability of the modified Atkins diet in adults with pharmacoresistant epilepsy: a prospective observational study.* Epilepsia, 2011. **52**(4): p. 775-80.
113. Kossoff, E.H., et al., *A prospective study of the modified Atkins diet for intractable epilepsy in adults.* Epilepsia, 2008. **49**(2): p. 316-9.

114. Sharma, S., et al., *Evaluation of a simplified modified Atkins diet for use by parents with low levels of literacy in children with refractory epilepsy: A randomized controlled trial.* Epilepsy Research, 2016. **127**: p. 152-159.
115. Vaccarezza, M.M., et al., *Treatment of refractory epilepsy with the modified Atkins diet.* Archivos argentinos de pediatria, 2014. **112**(4): p. 348-51.
116. Sharma, S., et al., *Use of the modified Atkins diet for treatment of refractory childhood epilepsy: A randomized controlled trial.* Epilepsia, 2013. **54**(3): p. 481-486.
117. Chen, W. and E.H. Kossoff, *Long-Term Follow-Up of Children Treated With the Modified Atkins Diet.* Journal of Child Neurology, 2012. **27**(6): p. 754-758.
118. Barzegar, M., I. Poupak, and M. Ebrahimi-Mameghani, *Modified Atkins Diet for intractable childhood epilepsy.* Iranian Journal of Child Neurology, 2010. **4**.
119. Kang, H.C., et al., *Use of a modified atkins diet in intractable childhood epilepsy.* Epilepsia, 2007. **48**(1): p. 182-186.
120. Kossoff, E.H., et al., *A modified Atkins diet is effective for the treatment of intractable pediatric epilepsy.* Epilepsia, 2006. **47**(2): p. 421-424.
121. Weber, S., et al., *Modified Atkins diet to children and adolescents with medical intractable epilepsy.* Seizure-European Journal of Epilepsy, 2009. **18**(4): p. 237-240.
122. Kossoff, E.H., J.L. Borsage, and A.M. Comi, *A pilot study of the modified Atkins diet for Sturge-Weber syndrome.* Epilepsy Research, 2010. **92**(2-3): p. 240-243.
